# Allometric growth and development of organs in ballan wrasse (*Labrus bergylta* Ascanius, 1767) larvae in relation to different live prey diets and growth rates

**DOI:** 10.1242/bio.017418

**Published:** 2016-07-15

**Authors:** Maren Ranheim Gagnat, Per-Arvid Wold, Tora Bardal, Gunvor Øie, Elin Kjørsvik

**Affiliations:** 1 Norwegian University of Science and Technology (NTNU), Dept. of Biology, Center for Fisheries and Aquaculture, Trondheim N-7491, Norway; 2 Norwegian Food Safety Authority, Division Namdal, Verftsgata 48, Namsos 7800, Norway; 3 SINTEF Fisheries & Aquaculture, Dept. of Marine Resources Technology, Trondheim N-7465, Norway

**Keywords:** Ballan wrasse, *Labrus bergylta*, Allometric growth, Larval growth, Organ development, Start feeding

## Abstract

Small fish larvae grow allometrically, but little is known about how this growth pattern may be affected by different growth rates and early diet quality. The present study investigates how different growth rates, caused by start-feeding with copepods or rotifers the first 30 days post-hatch (dph), affect allometric growth and development of nine major organs in ballan wrasse (*Labrus bergylta*) larvae up to experimental end at 60 dph. Feeding with cultivated copepod nauplii led to both increased larval somatic growth and faster development and growth of organ systems than feeding with rotifers. Of the organs studied, the digestive and respiratory organs increased the most in size between 4 and 8 dph, having a daily specific growth rate (SGR) between 30 and 40% in larvae fed copepods compared with 20% or less for rotifer-fed larvae. Muscle growth was prioritised from flexion stage and onwards, with a daily SGR close to 30% between 21 and 33 dph regardless of treatment. All larvae demonstrated a positive linear correlation between larval standard length (SL) and increase in total tissue volume, and no difference in allometric growth pattern was found between the larval treatments. A change from positive allometric to isometric growth was observed at a SL close to 6.0 mm, a sign associated with the start of metamorphosis. This was also where the larvae reached postflexion stage, and was accompanied by a change in growth pattern for most of the major organ systems. The first sign of a developing hepatopancreas was, however, first observed in the largest larva (17.4 mm SL, 55 dph), indicating that the metamorphosis in ballan wrasse is a gradual process lasting from 6.0 to at least 15-17 mm SL.

## Abstract (precis)

**Summary:** Allometric growth patterns of ballan wrasse were not affected by different diets, and larval functional development was found to be dependent on size and not age or growth rate.

## INTRODUCTION

Pelagic altricial fish larvae are among the smallest and most rapid-growing, free-living vertebrates. They are vulnerable, with organs that are not fully developed at hatching, and have an indirect development with a metamorphosis before reaching the juvenile phenotype (Blaxter, 1969; Osse et al., 1997; Balon, 1999). Larval growth and developmental rates are dependent on environmental factors and feed intake, and differences in growth and development has been found for many species when looking at fish in the wild and fish from different rearing conditions (Smith and Fuiman, 2004; Folkvord, 2005; Kjørsvik et al., 2009; Izquierdo et al., 2010; Karlsen et al., 2015; Øie et al., 2015). The high developmental plasticity, and the process of changing from a larval to an adult form through metamorphosis, affects both morphology, physiology, behaviour and the ecological niche of the fish (Pittman et al., 2013). A high plasticity is also present when larvae translate the environmental influence into somatic signals, such as somatogenesis, changes in morphology and in timing of the development (Pittman et al., 2013).

As the larvae develop, the body parts and organs most needed to enhance further growth and survival are prioritised, and these parts will have a higher relative growth and differentiation than other tissues. This type of growth pattern is called allometric growth, while a more isometric growth will generally be attained at metamorphosis (Fuiman, 1983; Osse and van den Boogaart, 2004; Sala et al., 2005). Studies of allometric growth patterns can give us more understanding about how the functional larval capacities develop in relation to environmental factors. Most studies of allometric growth in fish larvae have focused upon external characters in relation to general larval growth patterns and development (Fuiman, 1983; Gisbert et al., 2002; Nikolioudakis et al., 2010, 2014; Khemis et al., 2013; Martínez-Montaño et al., 2016), critical ontogenetic transitions (Gozlan et al., 1999; Simonovíc et al., 1999) and to the development of locomotion, swimming capacity and feeding behaviour (Osse and van den Boogaart, 1999). These studies often find two different shifts (inflection points) in relative growth, correlated to morpho-functional changes during the larval development.

Allometric growth studies of larval internal organs in *Dentex dentex* and *Psetta maxima* have demonstrated an especially rapid initial relative growth of digestive organs, later followed by a surge in trunk musculature (Sala et al., 2005). In carp (*Cyprinus carpio*), the liver, pancreas and trunk muscles had a positive allometric growth during the larval development (Alami-Durante, 1990). Very few have studied comparable allometric growth patterns in fish from different environments. Gozlan et al. (1999) compared external characteristics in *Chondrostoma toxostoma* larvae from the laboratory and from the field. In addition to larval growth differences, they found that the shifts in relative growth marking the transition to metamorphosis (or ontogenetic thresholds) occurred at a significantly smaller size for laboratory reared fish than for wild fish. However, how the relative growth and development of larval organ tissues may be affected by different growth rates and diet quality is not well known.

The ballan wrasse (*Labrus bergylta* L.) is a typical marine pelagic fish larva, with a very immature digestive system at the start of first feeding. It has a simple and rather undifferentiated gut (Dunaevskaya et al., 2012) with a low production of digestive enzymes (Hansen et al., 2013). The synthesis of pancreatic digestive enzymes increases quickly as the larval growth increases in pelagic larvae (Hoehne-Reitan and Kjørsvik, 2004). This species is also stomachless during its whole life. The ballan wrasse is used in salmon (*Salmo salar*) farming to de-louse salmon in the cages, and the interest in cultivation of this species is high (Ottesen et al., 2008; Skiftesvik et al., 2013). Like for most marine species, the commercial larval rearing is based upon cultivated, enriched rotifers and *Artemia* up to weaning. The pelagic larval natural diet consists mainly of zooplankton (copepods), and it is well known that marine fish larvae fed natural prey have a much faster somatic growth and survival, in addition to improved functionality and less deformities compared to those fed rotifers and Artemia (Witt et al., 1984; Næss et al., 1995; Shields et al., 1999; Imsland et al., 2006; Kortner et al., 2011; Karlsen et al., 2015). This is also observed for the ballan wrasse (Øie et al., 2015), and the growth differences is caused by a more beneficial nutrient composition in the copepods (Evjemo et al., 2003; van der Meeren et al., 2008; Karlsen et al., 2015; Øie et al., 2015).

For a fish produced for its ability to function well in a salmon cage, it would be imperative to understand how such differences in diet quality affects the functional development, growth mechanisms and general quality of the fish during commercial production. Also, information on the allometric growth of different organ groups may contribute to a better understanding of critical points during the larval development. Knowledge about early development and ontogeny of ballan wrasse is still very limited, and more information about its functional development, relative growth pattern, and nutritional requirements is needed.

Our aim was to determine the organ allometric growth pattern in developing ballan wrasse larvae, in order to understand the size-related adaptations and priorities during the larval stage. We also wanted to evaluate whether different somatic growth rates due to diet quality (copepods or rotifers) affected the allometric relative growth rates of internal organs, and thus the possible functional development of these larvae. To our knowledge, this is the first study to compare the effect of different diets on organ volume growth and allometric growth in fish larvae.

The characteristics of the larval diets and somatic growth of these same larvae are presented and discussed in Øie et al. (2015), where it was demonstrated that feeding with copepods during the first 30 days post-hatching (dph) led to an increased somatic growth compared to feeding with rotifers.

## RESULTS

### Larval growth and developmental stages

Feeding larvae with copepods resulted in a significantly better growth than feeding with rotifers, as shown for the dry weight (DW) and standard length (SL) in Table 1. Larvae from the Cop and Cop7 treatments had a significantly higher DW and SL than larvae from the RotMG and RotChl treatments at the end of the experiment (60 dph). The RotMG and RotChl larvae had similar growth pattern throughout the whole experiment (Table 1).

**Table 1.**
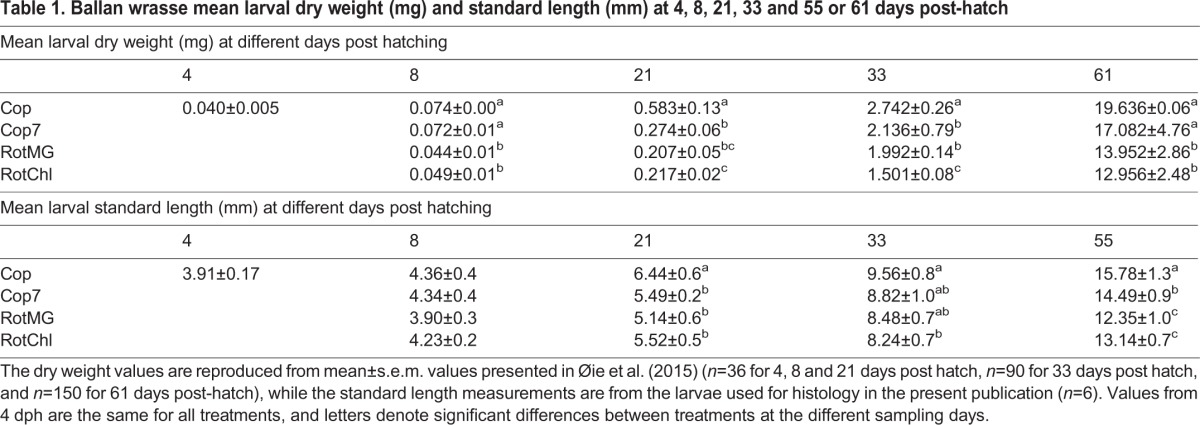
**Ballan wrasse mean larval dry weight (mg) and standard length (mm) at 4, 8, 21, 33 and 55 or 61 days post-hatch**

The yolk sac was visible up to 8 dph (100 day degrees post hatch; d^0^) in larvae from all treatments, after which the larvae switched to solely exogenous feeding (preflexion larvae). The Cop larvae developed faster than the other larvae, and had reached flexion larval stage at 13 dph (165 d^0^). At 21 dph (295 d^0^), all Cop larvae with a SL above 6.1 mm had developed into a post flexion larvae (4 out of 6 larvae). For the Cop7, RotMG and RotChl treatment, the preflexion larval stage lasted up to 18 dph (225-240 d^0^), followed by a transition to flexion larvae. All observed larvae had reached postflexion stage by 33 dph (475-490 d^0^), regardless of treatment.

### Organ development

**Fig. 1.**
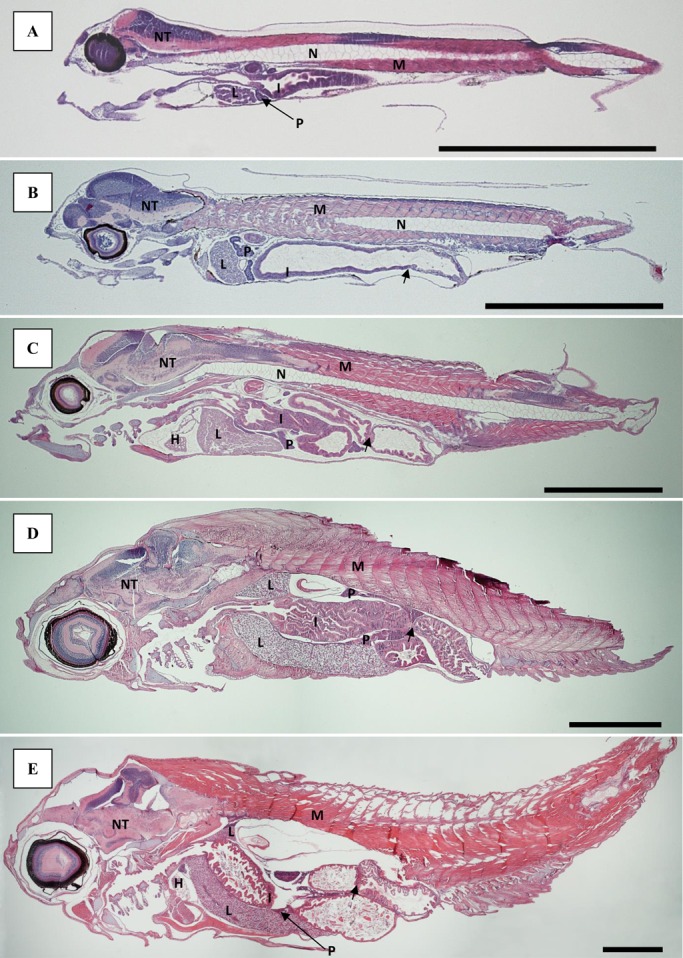
**Longitudinal sections of ballan wrasse larvae.** Sections shown at 4 (A), 8 (B), 21 (C), 33 (D) and 55 (E) days post-hatch stained with H&E. The valve separating the intestine into a prevalvular and postvalvular part is marked with a small arrow in B-E. Scale bar=1 mm. H, heart; I, intestine; L, liver; M, muscle; N, notochord; NT, nerve tissue; P, pancreas.

Longitudinal sections describing the general appearance of 4, 8, 21, 33 and 55 days old larvae are presented in Fig. 1A-E.

**Fig. 2.**
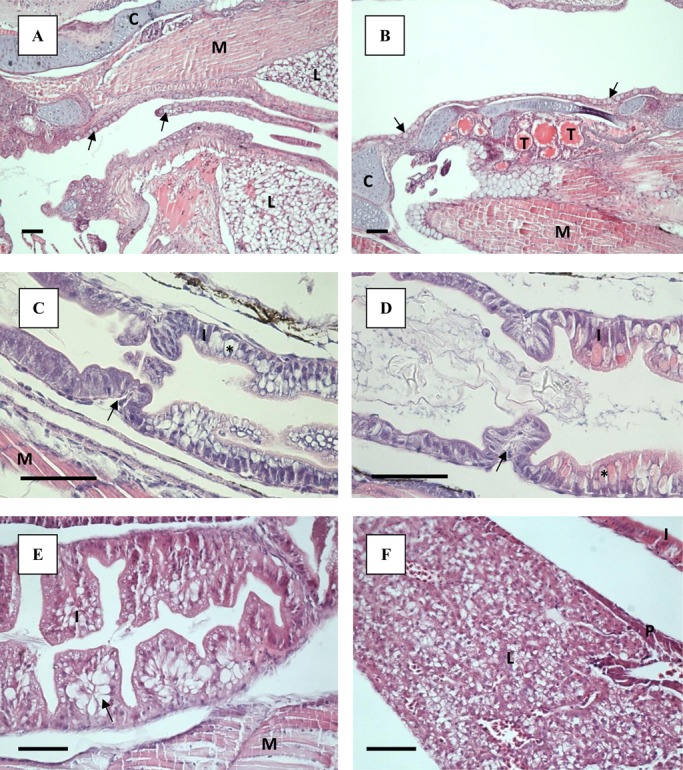
**Light microscopy sections of ballan wrasse larvae stained with H&E.** Scale bar=50 µm. (A) Longitudinal folds in oesophagus of a Cop larvae at 33 dph. Goblet cells pointed out by arrows. (B) Thyroidea (T), located in the centre of the lower jaw in larvae from the Cop treatment, 33 dph. Goblet cells pointed out by arrows. (C) Pre- and postvalvular intestine of Cop larvae at 8 dph. The different parts of the intestine are separated by a valve (arrow). Example of a supranuclear vacuoles is market with asterisk (*). (D) Pre- and postvalvular intestine of RotMG larvae at 8 dph. The different parts of the intestine are separated by a valve (arrow). Example of a supranuclear vacuoles is market with asterisk (*). (E) Lipid deposits in prevalvular intestine of RotMG larvae at 33 dph (arrow). (F) Liver and pancreas of Cop larvae at 55 dph. C, cartilage; I, intestine; L, liver; M, muscle; P, pancreas; T, thyroidea.

No goblet cells were present in the simple cuboidal epithelium lining the oesophagus at 4 dph. By 8 dph, small goblet cells with neutrally stained content had started to appear at the first narrowing of the pharynx, regardless of treatment. More goblet cells had appeared by 21 and 33 dph (Fig. 2A), also in the tissue lining the buccal cavity, and the content, had turned acidic and had a bright blue colour (H&E staining). By 33 dph, extensive longitudinal folds appeared along the length of the oesophagus, leading down towards the valve separating the oesophagus from the intestine (Fig. 2A). Primordial thyroid follicles were visible in the centre of the lower jaw by 8 dph. The colloid had a granular appearance. The follicles increased in number and size by 21 dph, and no difference was observed between the treatments. By 21 dph, vacuole-like spaces were occupying the periphery of the colloid, and by 33 dph the colloid appearance had changed from granular to smooth (Fig. 2B).

At 4 dph the intestine was separated by a thick valve into two regions; a pre-valvular and a post-valvular part. The enterocytes at both sides of the valve resembled each other in appearance, and the brush border was present. By 8 dph, the intestine had started to differentiate, with a pre-valvular region without goblet cells and a post-valvular region containing cells with vacuoles of varying sizes (Fig. 2C,D). The supranuclear vacuoles obtained no staining with Alcian Blue-PAS (i.e. they did not contain mucins), and the acidophilic content (Fig. 2D) were characterised as protein inclusion bodies. The intestinal wall of the larvae fed copepods appeared thicker and more developed compared to the RotMG and RotChl larvae at 8 dph (Fig. 2C,D). Long, longitudinal villi had appeared in the pre- and post-valvular intestine by 21 dph, increasing the intestinal surface (Fig. 1C,D). By 21 dph, goblet cells containing acidic mucins had also appeared in larvae from all treatments, mainly in the post-valvular intestine. In addition, supranuclear vacuoles were still present in the post-valvular intestine. During the *Artemia* phase, clusters of lipid deposition were observed in the enterocytes of the pre-valvular intestine in larvae from all treatments (Fig. 2E). These were located from the mid to the posterior part of the intestine, at the basal side of the cell. Goblet cells and vacuoles were present in the post-valvular intestine both at 33 and 55 dph. Coiling of the intestine occurred at a larval SL of 5.4 mm, regardless of treatment.

A large portion of yolk still remained at 4 dph, with the yolk sac being connected to the lower, anterior part of the liver. The hepatocytes were round and with central nuclei. No glycogen was observed in the hepatocytes prior to first feeding. Nearly all the yolk was resorbed at 8 dph. A varying degree of vacuolisation of the liver tissue between individuals was observed at 8 dph, with the vacuoles also varying in size. No pattern regarding larval size or treatment was observed, and at 21 dph no difference in degree of liver tissue vacuolisation was observed any more. By 33 dph, the liver had spread out ventrally, along the length of the abdominal cavity, and was also visible dorsally to the intestine (Fig. 1D). The pancreas was developed by 4 dph, and both endocrine (islet of Langerhans) and exocrine pancreatic tissue was observed. Triangular exocrine pancreatic acinar cells were gathered with their apices together. The nucleus was located at the base of the triangle cell, while zymogen granules were present in the apical part of the cell at 4 dph. The pancreas increased in size up to 21 dph, and in the largest larvae it spread throughout the whole abdominal cavity (Fig. 1C,D). At 55 dph the pancreas had started to stretch out along the lengths of the liver, with the largest larva having small pockets of exocrine pancreatic tissue incorporated into the liver (Fig. 2F).

### Volumetric tissue growth

Four-day-old larvae had an average total V_T_ of 0.075 mm^3^, increasing to close to 0.2 mm^3^ at 8 dph for larvae fed copepods (treatments Cop and Cop7) and close to 0.1 mm^3^ for larvae fed unenriched and enriched rotifers (treatments RotMG and RotChl) (Table 2). Larvae feeding on copepods continued to have significantly higher total V_T_ than those feeding on rotifers at 21 dph; the Cop larvae with an average total V_T_ of 1.522 mm^3^, more than double the volume of larvae from the other treatments which ranged from 0.59-0.69 mm^3^ (Table 2). At 55 dph, 25 days after all groups were transferred to the same diet (*Artemia*), the Cop larvae still had significantly larger total V_T_ than the RotMG and RotChl larvae, having on average increased their V_T_ 276 times since 4 dph compared to 132 and 170 times for the RotMG and RotChl larvae, respectively. The same tendency could be observed for the Cop7 larvae, although they were only significantly larger than the RotMG larvae at 55 dph.

**Table 2.**
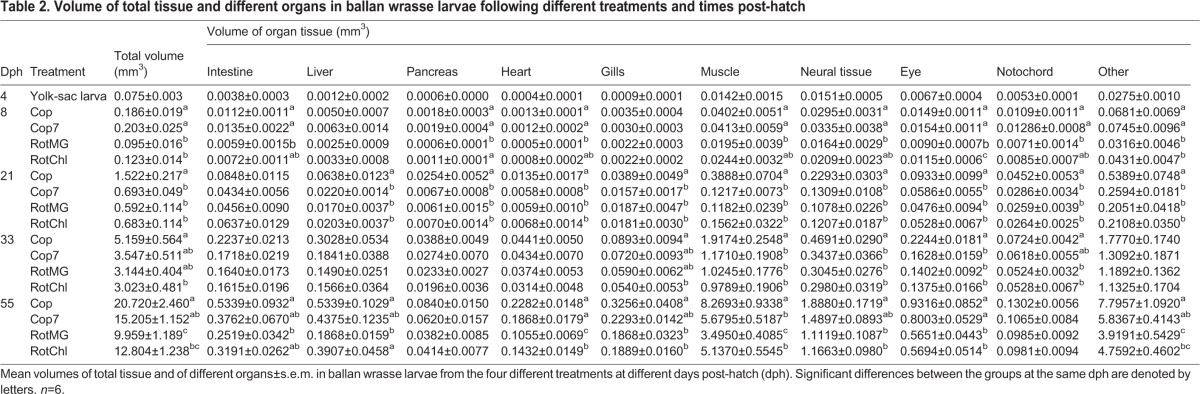
**Volume of total tissue and different organs in ballan wrasse larvae following different treatments and times post-hatch**

The relative volume of organ tissue (RV_T_), expressed as the percentage of the total volume of tissue, varied as the larvae developed. The RV_T_ of intestine, pancreas and liver was at 5.1, 0.8 and 1.5% respectively at 4 dph (Table 3). These organs had an increase in RV_T_ with increasing larval size which peaked at 21 dph for the intestine and pancreas, and at 33 dph for the liver, after which the RV_T_ decreased. Muscle and neural tissue were the two largest organ groups at 4 dph, with a V_T_ of respectively 0.0142 and 0.0151 mm^3^ (Table 2). This represented a RV_T_ at 4 dph close to 20% for both organ groups (Table 3). Muscle was the only organ group continuously increasing in RV_T_ throughout the experimental period, and went from values close to 20% of the larval total volume at 4 dph to between 35 and 40% at 55 dph (Table 3). At 21 dph, the Cop larvae had a significantly higher RV_T_ of muscle than larvae from the other treatments, while the Cop7 larvae had experienced a small drop in RV_T_ of muscle after switching feed from copepods to rotifers (Table 3). The RV_T_ for neural tissue, eyes and notochord decreased throughout the larval development (Table 3).

**Table 3.**
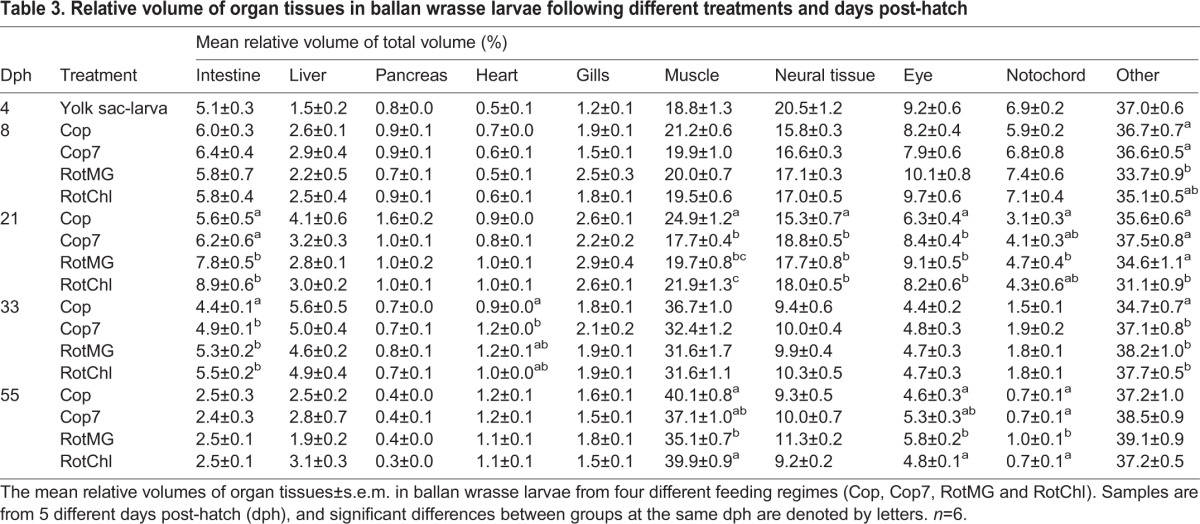
**Relative volume of organ tissues in ballan wrasse larvae following different treatments and days post-hatch**

In general, organs such as the intestine, liver, pancreas, heart, gills and muscle were the ones with the greatest increase in volume up to 21 dph. The tissue mass increased especially fast from 4 to 8 dph for those larvae receiving copepods as feed, and a daily SGR between 30 and 40% was observed for the liver, heart and gills (Table 4). An especially low tissue growth rate was observed after Cop7 larvae switched feed from copepods to rotifers, indicating that the larvae did not accept the new prey well (Table 4). During the transition period to *Artemia* feeding (21-33 dph), all groups had a similar specific tissue growth rate, with the muscle tissue growth rate being especially high during this period; close to 30% regardless of larval treatment (Table 4). The average daily SGR in total tissue varied from 9.5 to 11% for the period from 4 to 55 dph, with Cop larvae having the average highest growth and RotMG larvae the lowest.

**Table 4.**
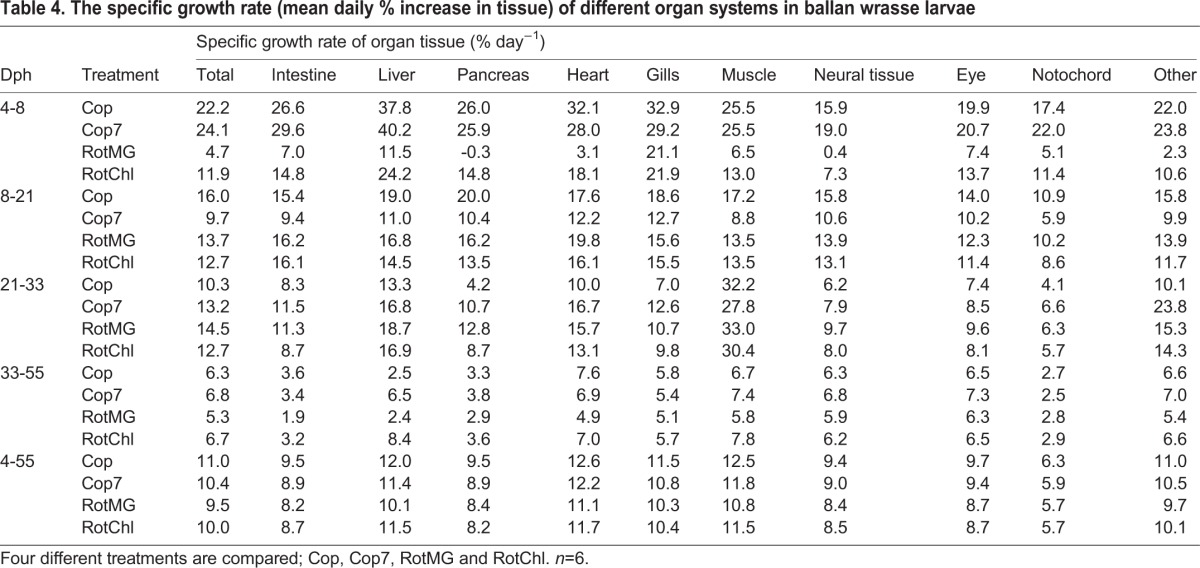
**The specific growth rate (mean daily % increase in tissue) of different organ systems in ballan wrasse larvae**

### Allometric tissue growth patterns

**Fig. 3.**
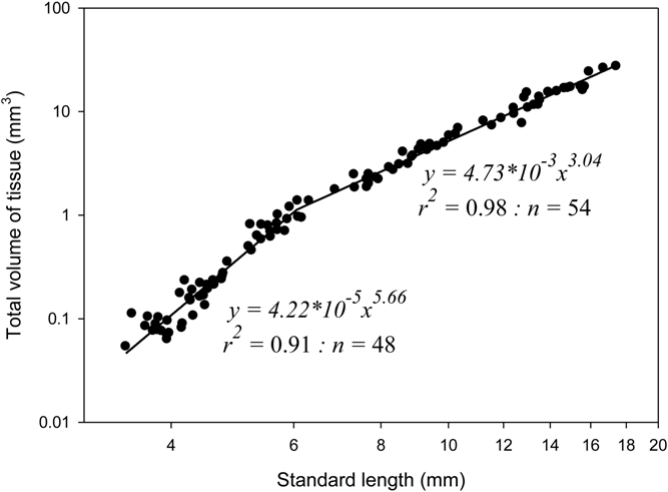
**The relationship between standard length and total volume of all tissue (mm^3^) for ballan wrasse larvae.** Each point represents values from individual larvae. Data collected from 4-, 8-, 21-, 33- and 55-day-old fish larvae. The growth is initially positive allometric (b=5.66), followed by near isometric growth (b=3.04) after an inflexion point at 6.1 mm.

No significant difference was found between the larval groups for any of the organ V_T_ measurements or total larval V_T_ when adjusting for larval length (measured by a one-way ANCOVA), and data from all the larvae were pooled for the allometric tissue growth patterns. A positive allometric increase (b=5.66) in larval total V_T_ was observed up to a larval SL of 6.1 mm, after which the growth became isometric (b=3.04) (Fig. 3).

**Fig. 4.**
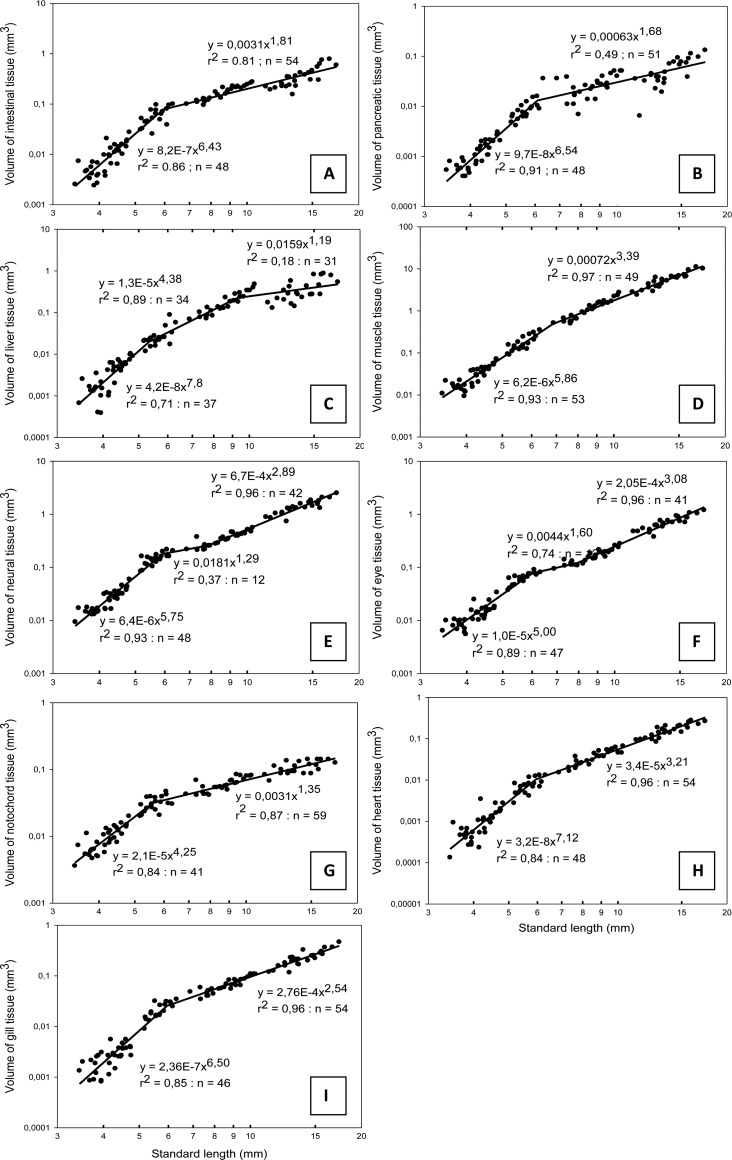
**Allometric growth equations and relationship for the standard length of ballan wrasse larvae and the volume of intestine, pancreas, liver, muscle, neural tissue, eyes, notochord, heart and gill tissue during early stages of development.** Each point represents measurements from a single larva. (A) intestine, (B) pancreas, (C) liver, (D) muscle, (E) neural tissue, (F) eyes, (G) notochord, (H) heart, (I) gill tissue.

The intestine and pancreas had a similar development throughout the whole period, with a highly positive allometric growth followed by a highly negative allometric growth after the inflexion points at a SL of respectively 6.0 mm and 6.1 mm (Fig. 4A,B). The growth coefficient of the liver was initially positive (b=7.80), becoming less positive (b=4.38) at a SL of 5.3 mm, followed by negatively allometric (b=1.19) after reaching 9.3 mm SL (Fig. 4C). Skeletal muscle tissue had a positive allometric growth with an initial growth coefficient of 5.86 (Fig. 4D) up to 6.9 mm SL, and thereafter close to isometric growth (b=3.39). The growth of neural tissue could be divided into three periods; with positive allometric growth up to 5.9 mm SL (b=5.75), followed by a period of negative allometric growth (b=1.29), before the growth became close to isometric at 7.9 mm SL (Fig. 4E). Eye tissue showed a similar pattern as for neural tissue (b between 1.6 and 5.0) with inflection points at 6.0 and 8.0 mm SL (Fig. 4F), while the notochord had initial positive allometric growth (b=4.25) becoming negative (b=1.35) after the inflexion point at 5.6 mm SL (Fig. 4G). Heart tissue had an initial positive allometric growth (b=7.12) (Fig. 4H), which changed to isometric around 6.0 mm SL. The initial positive allometric growth of gill tissue (b=6.50) became slightly negative (b=2.54) after the inflexion point at 6.0 mm SL (Fig. 4I). A summary of the different organ growth coefficients and larval SL at inflexion points can be seen in Table 5, where most of these organs had inflexion points close to a larval SL of 6.0 mm (<20 dph for copepod fed larvae). The neural tissue (including the eyes) had a second inflexion point around 8.0 mm SL, when they changed from negative allometry to isometric growth, and the latest inflexion point occurred when the liver switched to a negative allometry at 9.3 mm SL (30-35 dph).

**Table 5.**
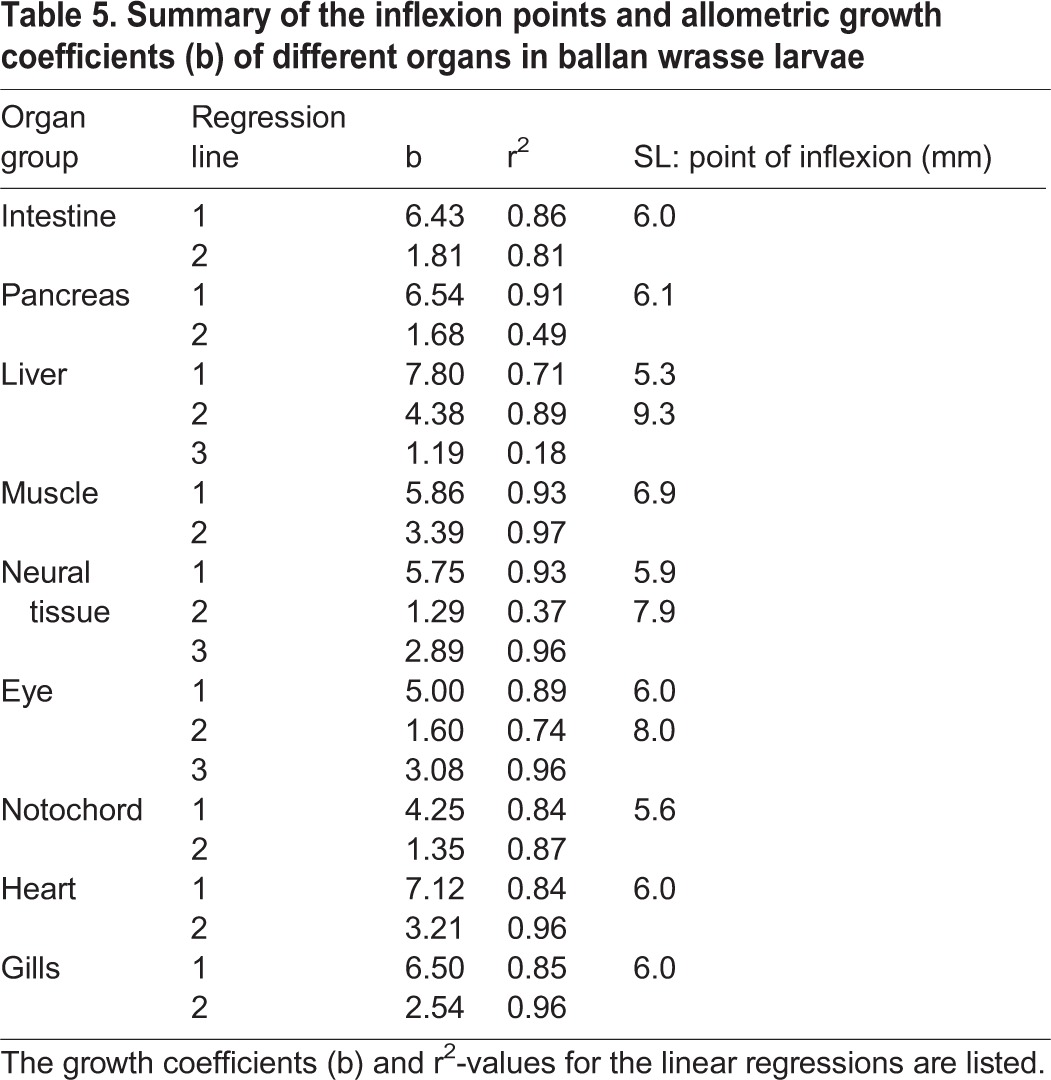
**Summary of the inflexion points and allometric growth coefficients (b) of different organs in ballan wrasse larvae**

Similar calculations of allometric growth coefficients and inflection points of the different organs were also made in relation to the total tissue volume V_T_ (see Fig. S1). Here the main inflexion points were also located around 6.0 mm SL, and the development showed similar allometric growth patterns as those related to SL. However, in relation to the total V_T_, the skeletal muscle and heart had a constant weak positive allometric growth during all stages, and the eye tissue had a constant negative allometric growth coefficient during the whole time.

## DISCUSSION

### Larval growth in relation to age

Using intensively cultivated copepod nauplii as feed for ballan wrasse larvae the first part of the start-feeding period resulted in a long-lasting increased somatic growth compared to feeding with rotifers. The copepod-fed larvae were significantly larger than those fed rotifers up to the end of the experiment (60 dph), and this occurred both when copepods were supplied up to 30 dph or only for the first 7 days (Øie et al., 2015). The copepod fed larvae also had a higher survival and better tolerance towards handling stress than those fed rotifers. A positive effect on marine larval somatic growth caused by feeding on copepods has also been found in species such as Atlantic cod (*Gadus morhua*) and Atlantic halibut (*Hippoglossus hippoglossus*) (Næss et al., 1995; Shields et al., 1999; Evjemo et al., 2003; Imsland et al., 2006; Koedijk et al., 2010; Busch et al., 2011; Karlsen et al., 2015). This has been attributed to a higher fraction of essential fatty acids (EFAs) and polar lipids, and a greater amount of proteins and free amino acids (FAAs) in the copepods (Evjemo and Olsen, 1997; Bell et al., 2003; Evjemo et al., 2003; Tocher et al., 2008; van der Meeren et al., 2008; Olsen et al., 2014).

In our experiment, exogenous feeding started at 4 dph, and the yolk sac was close to depletion at 100 d^0^ (8 dph, 4.2-4.3 mm SL) in larvae from all treatments. This is similar to observations by Ottesen et al. (2012), who fed the larvae enriched rotifers followed by *Artemia* (similar to our RotMG treatment). While Ottesen et al. (2012) observed transition to the flexion stage at 340 d^0^ (26 dph), we observed this earlier for all our larvae; from 165 d^0^ (13 dph, Cop treatment), and at 225-240 d^0^ for all other groups (18 dph), reflecting a faster growth in our larvae. This growth and developmental difference between larvae given similar diets could be expected, as we used a slightly higher temperature than Ottesen et al. (2012) during the whole experiment (1-2°C higher). The ballan wrasse flexion stage lasts until formation of the first caudal fin rays separate from the dorsal fin rays in the margin of the finfold, and all fins are developed. The larval body is fully pigmented, except the urostyle region which becomes pigmented during the postflexion stage (Ottesen et al. (2012). Also the transition to postflexion was observed earlier, with two thirds of the Cop-larvae (those above 6.1 mm) changing to the postflexion stage already at 295 d^0^ (21 dph), compared to 470 d^0^ (ca 34 dph) for the larvae in the experiment of Ottesen et al. (2012). However, larval size (SL) at transition from flexion to postflexion at 5.9-6.1 mm SL, matched well with the observations of Ottesen et al. (2012).

The larval size (SL) correlated positively linearly to the larval total tissue volume (V_T_), and the change in growth pattern from initially positive allometric growth to isometric growth occurred at a mean size of 6.1 mm SL. This is around the same length as the transition from flexion to postflexion larvae occurred (5.9-6.1 mm SL). During this two-phased pattern of increase in total V_T_, we found an initial slow increase in total V_T_, and a sharp rise in tissue volume after the inflexion point; compared to the 4-day-old larvae, the copepod-fed larvae increased their total tissue volume 20 times by 21 dph, whereas the volume had increased 276 times by the end at 55 dph (see Table 2).

### Organ tissue development and relative growth

At the start of the exogenous feeding (4 dph), the alimentary canal was differentiated into buccopharynx, oesophagus, pre- and postvalvular intestine and rectum. A well-defined liver and a pancreas were present in the ballan wrasse larvae, and no glycogen was observed in the liver at 4 dph. In general, the ontogeny of the digestive system followed the pattern described by Dunaevskaya et al. (2012). However, we found a clear brush border from 4 dph, while Dunaevskaya et al. (2012) first observed this from 7 dph. Supranuclear vacuoles with acidophilic content were observed in the post-valvular intestine by 8 dph. These are typical for the larval pinocytotic activity in the hindgut, with accumulation and intracellular digestion of protein (Watanabe, 1981, 1984). In this early preflexion stage, the prevalvular intestinal wall of the copepod fed larvae appeared to have increased more in height and looked more developed at 8 dph, compared to the RotMG and RotChl larvae. This was supported with a higher V_T_ for the intestine at 8 dph for the larvae fed copepods, and a higher SGR_T_ from 4 to 8 dph. Suboptimal diets or starvation is known to cause reduced height of the intestinal wall (Theilacker and Watanabe, 1989; Kjørsvik et al., 1991, 2011), and the rotifer fed ballan wrasse larvae clearly did not reach their potential for early growth and development in the transition phase (mixed feeding stage) from the yolk-sac stage to first-feeding larvae.

While there was little variation in the organ tissue structures between the different treatments throughout the development, the organ tissue growth varied remarkably in relation to larval age. The larvae receiving copepods had a daily total tissue SGR of 22-24% from 4 to 8 dph, which was twice as high, or more, as the ones receiving rotifers of any kind. As expected for early larval growth (Segner et al., 1994; Gisbert, 1999; Osse and van den Boogaart, 2004; Sala et al., 2005), the most important organs related to digestion and respiration, such as the liver, heart and gills, increased the fastest during this period. For larvae fed copepods, the daily SGR for these organs were between 30 and 40%, compared to 20% or less for the rotifer fed larvae. Our measured growth rate of the intestine, liver, pancreas, heart and gills continued to be higher than, or similar to the total somatic growth rate up to 21 dph, and all these organ groups had an initial positive allometric growth, eventually turning negative for all organs except for the heart.

However, compared to larval size (SL or V_T_), no difference was found in the relative growth of the larval organs. We thus found no effects in the relative organ growth pattern from diet quality, and our findings are thus supporting the emerging data and hypothesis that larval functional development is more dependent on size than on age (Hoehne-Reitan and Kjørsvik, 2004; Sæle and Pittman, 2010). The liver had an especially rapid allometric growth in the preflexion stage, and changed to a somewhat slower positive allometric growth from the transition to the flexion stage, demonstrating the early importance of this organ. This early rapid liver growth in the ballan wrasse coincides well with an observed sharp increase in transcripts of the liver enzyme Cyp7 A1, cholesterol 7α-hydroxylase during preflexion (Hansen et al., 2013), which is a key enzyme for conversion of cholesterol to bile acid in the liver. A second inflexion point (9.3 mm SL) to negative allometric growth was found for the liver during the postflexion stage, and small indentations of pancreatic tissue was only found in the largest larva at the end of the experiment (>17 mm SL), suggesting a development at this size towards the hepatopancreas mode found in juveniles (E.K., unpublished).

Also the pancreas and the intestine had an especially positive allometric growth initially, changing into negative allometric growth after inflexion points at respectively 6.1 and 6.0 mm SL. This coincided with the transition to the flexion stage. At around the same size (6-7 mm SL), Hansen et al. (2013) found a rapid increase in key pancreatic digestive enzymes gene expressions in ballan wrasse larvae fed rotifers.

Larvae of the common dentex (*Dentex dentex*) and turbot (*Psetta maxima*) have shown similar development, with the digestive organs having a fast relative growth during early development and the highest allometric growth coefficients (b>2) of all organ systems studied (Sala et al., 2005). A prioritised volume increase of the pancreas and liver was also observed during early development of common carp (*Cyprinus carpio* L.) larvae, where these organs, together with muscle tissue, were the only organs with positive allometric growth coefficients (b=1.19 for pancreas and liver, and 1.09 for muscle) (Alami-Durante, 1990).

Muscle and neural tissue represented the major proportion of the ballan wrasse larval total tissue at all days investigated. Muscle accounted for close to 20% of the total larval V_T_ at 4 dph, as did also the neural tissue. This is similar to what is reported for common dentex and common carp (Alami-Durante, 1990; Sala et al., 2005). As was also observed for those species, the ballan wrasse larvae had an increasing proportion of axial musculature and a decreasing proportion of neural tissue with increasing body mass. By 55 dph, the ballan wrasse axial muscular tissue represented between 35 and 40% of the larval total V_T_, while the neural tissue had stabilised at a RV_T_ close to 10%.

While total V_T_ for the copepod fed larvae increased 275 times between 4 and 55 dph (data from Table 2), the muscle tissue increased around 580 times during this period, demonstrating an extraordinary growth priority for muscle development. Most of the muscle volume increase occurred in larvae >9 mm SL, i.e. after the second liver inflexion point. While the SGR for most organs decreased after the preflexion period (up to 21 dph), muscle tissue had the highest growth rate from the flexion stage and onwards, growing at an average daily rate close to 30% between 21 and 33 dph regardless of treatment. This was a far higher growth rate than any of the other organs during the same period, demonstrating a prioritised increase in muscular tissue during this period. Muscle was also the only organ tissue found to continuously increase in RV_T_ up to 55 dph, and it had a biphasic growth pattern with an initial highly positive allometric growth (b=5.86) turning into a slightly positive allometric growth after the inflection point (b=3.39). In relation to the total V_T_, the increase in skeletal muscle was found to have a constant weak positive allometric growth throughout the whole experimental period (b=1.15). This difference found by comparing allometric growth against SL or total V_T_ is likely a result of the total V_T_ increasing faster up to 6.1 mm SL than increase in length. In other studies, the common carp and common dentex had a similar pattern of increase in muscular V_T_ with regard to total V_T_ as for the ballan wrasse, with the allometric growth being constant positive and with similar growth coefficients (b=1.10 for common dentex and 1.09 for common carp) (Alami-Durante, 1990; Sala et al., 2005). A biphasic growth pattern was also observed for the turbot, having a negative allometric increase in muscle V_T_ in the beginning (b=0.85) followed by a positive allometric growth later (b=1.26) (Sala et al., 2005).

A negative allometric growth of neural tissue and eyes is generally observed throughout larval development (Alami-Durante, 1990; Sala et al., 2005) and in adult fish (Oikawa et al., 1992; Schultz et al., 1999). While allometric growth of eye tissue was continuously negative in ballan wrasse in relation to the total V_T_, eyes had a three-phased growth pattern when compared to larval SL. A very high positive allometric growth coefficient was found up to 6 mm SL, then a brief negative period, before growing isometrically from around 8 mm SL. A similar growth pattern was observed for neural tissue compared to SL. The initial positive growth would reflect the importance of eyes and neural tissues in the earliest stages. Brain and eye tissue have a rapid development prior to hatching, and having highly developed sensory organs after hatching and at the time of first feeding is necessary for prey detection and capturing (Osse and van den Boogaart, 1999). Negative allometry of these organs is a well-known feature later in development, caused by the continuous growth of the fish throughout their lives (Kotrschal et al., 1998).

As reviewed by Pittman et al. (2013), fish larvae display a high plasticity in how they translate environmental influence into somatic signals, such as somatogenesis, changes in morphology and in timing of the development. At the end of our experiment, the mean total tissue volume of copepod fed larvae was more than twice that of the smallest group of rotifer fed larvae (20.7 vs 10 mm^3^), even though they had been receiving the same diet from 30 dph. In addition to somatic growth rates, the early diet quality was found to affect ballan wrasse larval mortality and functionality (Øie et al., 2015). The same result was also found for Atlantic cod (Øie et al., 2015). In addition the larvae fed copepods had a higher handling stress tolerance, and were found to be better predators, capturing prey more efficiently (Øie et al., 2015). A deficiency of dietary EFAs has been found to affect development of neural tissue and larval vision (Bell et al., 1995; Furuita et al., 1998; Sargent et al., 1999). For instance, Shields et al. (1999) found that feeding with zooplankton had a positive effect on the number of rods in the retina of halibut larvae as opposed to feeding with *Artemia*, and larval herring fed *Artemia* deficient in DHA experienced a loss of visual function and less effective prey predation (Bell et al., 1995). The differences observed in ballan wrasse larval ability to capture prey, stress tolerance and ultimately in the differences in survival and size at the end of the experiment could therefore be an effect from the first feed nutritional quality on the functionality of the sensory organs, or the interactions of these organs and the muscular tissue, which in turn could inflict long term effects for the larvae. For a species where the cultivation is dependent on the fish turning out to be a good louse predator, more studies should be performed with a focus on whether or not the early diet has any long term effect on the ability to locate and capture prey.

Although the earliest prey quality affected somatic growth rates, the developmental stages was clearly linked to larval size (SL) and not to age, and the allometric growth patterns of vital organs was not affected in the surviving larvae. The inflexion point of total V_T_ was at around 6.0 mm SL, at the same time as the transition to postflexion stage, and also where a change from highly positive allometric growth to negative occurred for most organs groups (intestine, pancreas, neural tissue, eyes, notochord and gills). In addition, we observed that the first ossification of vertebrae segments in ballan wrasse larvae occurred from a SL of 5.8 - 6.8 mm (E.K., unpublished data). Although a multi-character approach is recommended for determination of size at metamorphosis (Nikolioudakis et al., 2010), the fin development, vertebral ossification, and change in the general growth pattern from allometric to isometric has been listed as some of the key events in the process of metamorphosis (Osse et al., 1997; Kjørsvik et al., 2004). The developmental changes in these characters thus demonstrate that ballan wrasse metamorphosis is initiated at a SL close to 6.0 mm.

The end of metamorphosis could however not be determined from the allometric growth data in our study. A second inflexion point was observed between 8-9 mm SL, when eyes and neural tissues changed from negative allometry to isometric growth, and the liver went from positive to negative allometric growth. However, the juvenile ballan wrasse additionally has a fully ossified vertebra, ossified scales and a well-developed hepatopancreas (E.K., unpublished). We observed that the most active vertebral ossification period happened between 7 and 10 mm SL, and ossified scales first covered the body in larvae >15 mm SL (E.K., unpublished). In addition, we found signs of small pockets of exocrine pancreas incorporated into the liver only in the largest larva (17.4 mm SL). This implies that the metamorphosis in the ballan wrasse is a gradual process, lasting from around 6.0 mm SL and at least up to 15-17 mm SL. The observed differences in timing of allometric inflexion points, ossification of the vertebrae, squamation and development of a hepatopancreas emphasise the importance of using multifactorial analysis of allometric and morphological changes, as suggested by Nikolioudakis et al. (2010, 2014), as a tool for estimating the size at metamorphosis in ballan wrasse.

### Conclusions

The ballan wrasse larval somatic growth and the growth of different organ tissues were much faster with copepod nauplii compared to rotifers as a first-feeding diet. The organ SGR was especially rapid from 4 to 8 dph, where growth of digestive and respiratory organs was prioritised, while muscle growth was prioritised later, from 21 to 33 dph.

The allometric growth pattern in relation to larval size was not affected by the different diets and larval growth rates, and the total tissue volume correlated positively linearly to the larval SL. Ballan wrasse larval functional development was thus dependent on size and not on age or on growth rate.

A change in the allometric growth pattern of total tissue was found around 6.0 mm SL, and at this size the first inflexion points and major changes in allometric growth of the different organs also occurred. Based upon the shifting allometric growth coefficients and morphological features, we suggest that the metamorphosis process occurs in ballan wrasse in the size range from 6.0 to at least 15-17 mm SL.

## MATERIALS AND METHODS

The experimental and analytical work was performed at NTNU Centre of Fisheries and Aquaculture in Trondheim, Norway, and all work was carried out according to the EU Directive 2010/63/EU for animal experiments and Norwegian animal welfare legislation.

### The experiment

The ballan wrasse larvae (2 days post-hatch, dph) were supplied from Marine Harvest LABRUS (Øygarden, Norway). Upon arrival they were transferred to 100-litre cone-bottomed tanks with an estimated density of 84 larvae l^−1^. The temperature increased from 12 to 16°C during the first 22 days, and was kept stable thereafter. Four different feeding regimes were tested on the ballan wrasse larvae, with three replicate tanks of each treatment. The regimes varied in the type of live feed provided during the first 30 days, and addition of live feed started at 4 dph. Larvae from the ‘Cop’ treatment were fed exclusively with intensively cultivated *Acartia tonsa* nauplii fed a monoalgal diet of *Rhodomonas baltica* (Støttrup et al., 1986). Larvae from the ‘Cop7’ treatment were fed *A. tonsa* nauplii from 4 to 10 dph, with a transition to rotifers (*Brachionus ibericus*, Cayman) cultivated on DHA *Chlorella* (Chlorella Industry Co. Ltd, Tokyo, Japan) and short-term enriched (2 h) on Multigain (BioMar AS, Myre, Norway). Fish larvae from the ‘RotMG’ treatment received short-term enriched rotifers the whole period, while the ‘RotChl’ treatment had a diet consisting of rotifers without any short-term enrichment. Larval feeding occurred three times a day at a density of 12,000 l^−1^. This was increased to four times a day after 19 dph. All larval groups had a co-feeding period with *Artemia fransiscana* from 24 to 30 dph, before being fed *Artemia* exclusively up to 40 dph (density 3000 l^−1^). Weaning to a formulated diet (Nofima, Tromsø, Norway, size 600-800 µm) occurred between 40 and 50 dph, and from 51 dph each tank received a total of 10 g formulated feed per day. The experiment ended at 61 dph. More details regarding production and nutritional composition of the live prey, and the larval rearing, growth and survival, are presented in Øie et al. (2015).

#### Larval fixation and measurements

Larvae were randomly sampled and anesthetised using tricaine methanesulfonate (MS-222 Finquel^®^, Agent Chemical Laboratories Inc., Redmond, WA, USA). Larval sampling and analysis of somatic growth was done according to Øie et al. (2015).

Larval developmental stage was determined based on external morphological features and changes occurring during ballan wrasse larval development, as described by Ottesen et al. (2012), where the larval development is classified into four stages; yolk-sac larva, preflexion larva, flexion larva and postflexion larva. For the histological analysis, the fish larvae were fixated in 4% formaldehyde in phosphate buffered saline (pH 7.4; Apotekproduksjon AS, Oslo, Norway) and stored cold (4°C) in glass vials.

For tissue volume analysis, six larvae were analysed from each treatment on the selected sample days (4, 8, 21, 33 and 55 dph). The larval standard length (SL) was measured on all fixed larvae. The larvae were then embedded in paraffin (Tissue-Tek^®^ III Embedding wax, Sakura Finetek, Alphen aan den Rijn, The Netherlands), cut into 4-µm-thick longitudinal sections (Jung Autocut 2055, Leica Microsystems, Wetzlar, Germany) and stained with Mayer's hematoxylin solution and eosin 0.5% aqueous Y-solution (Merck, Damstadt, Germany) (H&E staining). Alcian Blue-PAS staining (Alcian Blue solution and PAS-staining kit, Merck, Damstadt, Germany) was used to check for glycogen and mucins. Sections were studied live using a Zeiss Axioskop 2 plus microscope (Zeiss Inc., Oberkochen, Germany) equipped with a JVC TK-C1381 colour video camera (JVC, Yokohama, Japan).

Tissue volumes were estimated by the Cavalieri method (Howard and Reed, 1998), using CAST 2 (Olympus Inc., Ballerup, Denmark) to apply a point grid (Michel and Cruzorive, 1988; Mayhew, 1991; Howard and Reed, 1998). Points touching any tissue were registered as hits in its respective category. Points not touching tissue, or touching the lumen of the buccopharyngeal cavity, digestive tract or swim bladder, were not registered as hits (Sala et al., 2005). The volume of tissue (V_T_) of nine different organs was determined: intestine, liver, pancreas, heart, gills, muscle, nerve tissue (brain+spinal cord), eye and notochord, in addition to a tenth category named ‘other’, consisting of all tissues not covered by the previous categories (e.g. cartilage, kidney, oesophagus, buccal cavity, and swim bladder). Together these 10 categories covered all tissues in the fish larvae, which together made up the larval reference volume (Howard and Reed, 1998). The transition from oesophagus to intestine was determined at the valve separating the simple cuboidal epithelium of the oesophagus from the simple columnar ciliated epithelium of the intestine.

For the measurements of larvae from 4, 8 and 21 dph, the distance between each section studied was determined so that every developed organ would be present in at least five studied sections. The interval between the sections from 33 and 55 dph was determined as the minimal distance keeping the measurement error of one of the most irregular organs, the pancreas, below 10%. Details regarding the sectioning, point grid and magnification used are summarised in Table 6. The V_T_ was calculated from the equation V_T_=∑A×(E+C), where A represents the summation of the measured area section, E the thickness of the section and C the distance between the measured sections.

**Table 6.**
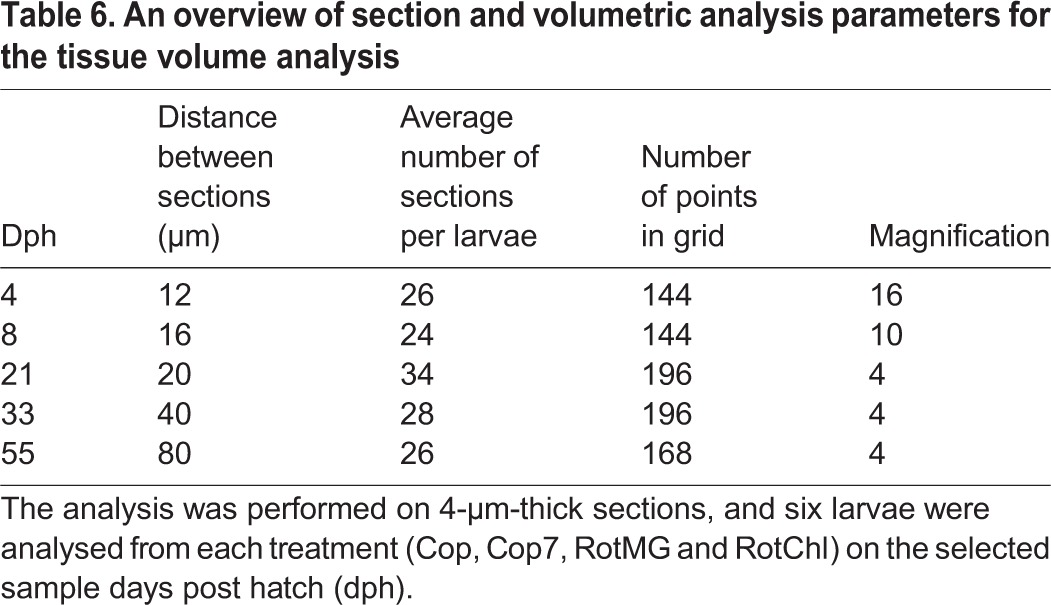
**An overview of section and volumetric analysis parameters for the tissue volume analysis**

### Statistical analysis of data

Statistical testing on percentage and volume fraction values was done on Arc sine transformed data. All data were normally distributed (Shapiro–Wilk test), and difference of means was tested using a One-way ANOVA. This was followed by the *post hoc* test Student–Newman–Keuls if there were homogeneity of variance, and Dunnett T3 if there were not. The homogeneity of variance was tested with a Levene test.

A one-way ANCOVA for independent samples was run to determine if there were any size related (SL) difference in organ V_T_ between the treatments. Since no difference was detected, the data was pooled for the allometric growth studies. Allometric growth was calculated as a power function of total V_T_ and SL using non-transformed data: y=ax^b^; where y is the measured character (organ V_T_), a the intercept, x the total V_T_ or SL, and b the growth coefficient (Fuiman, 1983). A growth coefficient of 1 indicates isometric growth, while one grater or less than 1 indicates positive or negative allometric growth respectively when comparing volume to volume. When comparing volume to SL, a growth coefficient of 3 equals isometric growth (Gisbert, 1999). To determine whether the data was best described as a piecewise linear function or a simple linear regression, the possible inflexion points had to be found. The piecewise linear function resulting in the best fit for the data was determined in the program Matlab with the algorithm (Broken Stick Regression) using POLYFIT and FMINSEARCH to determine the location of the inflexion points. The number of inflexion points was increased from 0 to 3, and the calculations were done on log-transformed data. After determining the linear functions, a *t*-test (α=0.05, *n*–4 degrees of freedom, Eqns 1 and 2) was used to check if the slopes (b) of the subsequent linear pieces were significantly different from each other:
(1)
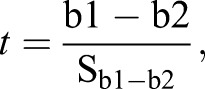

(2)
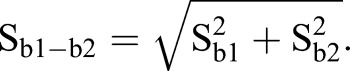


b1 and b2 are the slopes of the two lines tested against each other, and S_b1–b2_ is the standard error of the difference between the two slopes. S_b1_ and S_b2_ is the standard error of the different slopes (http://core.ecu.edu/psyc/wuenschk/docs30/CompareCorrCoeff.pdf). A *F*-test (*n*–4 degrees of freedom, Eqn 3) was used to determine whether the different piecewise linear functions were better fits than just using a linear regression. If several piecewise linear functions gave a better fit (and the *t*-test had given that all the subsequent slopes were significantly different from each other), the one with the highest *F*-value was used.
(3)
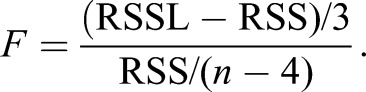
RSSL=the residual sum of squares of the linear regression and RSS=the residual sum of squares for the piecewise linear function (Vieth, 1989). A Pearson correlation coefficient was used to describe how well the data fit the linear relationship.
